# Diversity of the Tryptophanase Gene and Its Evolutionary Implications in Living Organisms

**DOI:** 10.3390/microorganisms9102156

**Published:** 2021-10-15

**Authors:** Bharath Reddy Boya, Prasun Kumar, Jin-Hyung Lee, Jintae Lee

**Affiliations:** School of Chemical Engineering, Yeungnam University, 280 Daehak-Ro, Gyeongsan 38541, Korea; bharathreddy2696@gmail.com (B.R.B.); prasunbiotek@gmail.com (P.K.); jinhlee@ynu.ac.kr (J.-H.L.)

**Keywords:** archaea, horizontal gene transfer, indole, marine organisms, phylogeny, tryptophanase

## Abstract

Tryptophanase encoded by the gene *tnaA* is a pyridoxal phosphate-dependent enzyme that catalyses the conversion of tryptophan to indole, which is commonly used as an intra- and interspecies signalling molecule, particularly by microbes. However, the production of indole is rare in eukaryotic organisms. A nucleotide and protein database search revealed *tnaA* is commonly reported in various Gram-negative bacteria, but that only a few Gram-positive bacteria and archaea possess the gene. The presence of *tnaA* in eukaryotes, particularly protozoans and marine organisms, demonstrates the importance of this gene in the animal kingdom. Here, we document the distribution of *tnaA* and its acquisition and expansion among different taxonomic groups, many of which are usually categorized as non-indole producers. This study provides an opportunity to understand the intriguing role played by *tnaA*, and its distribution among various types of organisms.

## 1. Introduction

Tryptophanase (TnaA) is a pyridoxal 5′phosphate-dependent enzyme that catalyses the hydrolytic β-elimination of tryptophan to indole, pyruvate, and ammonia, which all play unique roles within organisms and the environment [1,2,3]. Consequently, it is an important enzyme from the perspective of amino acid and nitrogen metabolism. Pyruvate is a key molecule that links carbon and nitrogen metabolic pathways, drives metabolic flux according to environmental conditions and helps to regenerate reducing equivalents under aerobic and anaerobic environments [1]. Re-routing of pyruvate toward gluconeogenesis is associated with the maintenance of enterohaemorrhagic *Escherichia coli* O157:H7 in bovine intestines [1]. The degradation of tryptophan, like other amino acids, results in the production of ammonia as a by-product, which in turn may participate in other biochemical reactions such as nitrogen cycle post excretion into the environment.

Indole is a unique compound with wide-ranging effects on many organisms [4,5] and its involvement as an effector molecule in prokaryotes and eukaryotes is intriguing. In prokaryotes, it serves as a signalling molecule that modulates cell division, virulence, and biofilm formation [5]. Antibiotic tolerance is a major menace in clinical medicine [6,7] and indole contributes to the resistance acting as a signalling molecule [5]. In addition, indole-producing bacteria can interfere with quorum sensing, biofilm formation, antibiotic tolerance, pigment production, and predation abilities of non-indole producing bacteria including pathogens [2,3,4,5,8,9].

In eukaryotes, indole and indole-derived compounds display a broader significance. In insects, indole is a cue to identify food, mating partners, and ovipositional sites [10,11,12,13]. Indole also participates in survival dynamics and is utilized in predator–prey interactions and plant defence mechanisms [14,15,16]. Fungi and several marine organisms can use indole to produce indole-derived compounds, such as pigments, alkaloids, Tyrian purple, and antifouling agents [17,18,19]. In vertebrates, including humans, tryptophan metabolism leads to the synthesis of indole moieties containing precursors of key mediators such as serotonin, melatonin, kynurenine, and tryptamine [20,21,22].

Thus, available evidence demonstrates that tryptophan metabolism plays vital roles in various living forms [23]. The ability of TnaA to conduct reverse α,β-elimination and β-substitution reactions has also attracted attention because the former can be used to synthesize tryptophan [24]. Furthermore, the ability of TnaA to synthesize tryptophan is important in higher eukaryotic hosts because it is used by gut microbiota to produce the rare amino acid tryptophan [1]. Therefore, we decided to study the lineage and diversity of the *tnaA* gene to identify the roles of indole and TnaA in organisms and biotechnological applications associated with variations in the active sites of TnaA, especially concerning the biosynthesis of hormones and alkaloids.

## 2. Materials and Methods

### 2.1. Sequence Data/Data Mining

The *tnaA* sequences (>1200 nts) of 221 isolates of 36 eukaryotes (Drosophila, hemichordates and marine organisms including octopus, sea anemone, corals, hermit crab), 41 archaea, 16 fungi, and 128 prokaryotes (Gram-positive and -negative bacteria) were investigated in the present study. These gene sequences were obtained from the NCBI database (https://www.ncbi.nlm.nih.gov/gene accessed on 8 October 2020). The following exclusion terms were used: putative protein; TnaA leader peptide (*tnaC*); tryptophan 2,3-dioxygenase; and hypothetical proteins; record removed; and large eukaryote sequences (with long ‘N’ stretches). Curation of *tnaA* sequences was performed manually to avoid redundant/partial or non-related sequences.

### 2.2. Phylogenetic Analyses

For the nucleotide sequence phylogenetic tree, the 221 *tnaA* nucleotide sequences were assembled and translated to their respective cDNA sequences and aligned using the multiple sequence alignment program ClustalX version 2.0.12. To estimate evolutionary distances, pairwise distances between species were calculated using the MEGA 11 package. A total of 221 *tnaA* ortholog sequences were analysed. Sequences were aligned with MEGA 11 and alignment errors were corrected using BioEdit 7.2. Evolutionary history was inferred using the Maximum Likelihood method and General Time Reversible model. Initial trees for the heuristic search were obtained automatically by applying Neighbor-Join and BioNJ algorithms to a matrix of pairwise distances estimated using the Maximum Composite Likelihood (MCL) approach, and then selecting the topology with superior log likelihood value. A discrete Gamma distribution was used to model evolutionary rate differences among sites (4 categories (+*G*, parameter = 1.0799)). The rate variation model allowed for some sites to be evolutionarily invariable. The analysis was conducted for 1000 bootstrap replications. The tree is drawn to scale, with branch lengths measured in the number of substitutions per site. This analysis involved 221 nucleotide sequences. There was a total of 32,115 positions in the final dataset. Evolutionary analyses were conducted in MEGA11.

For the amino acid sequence phylogenetic tree, the nucleotide sequences were translated using MEGA 11, assembled and aligned using the multiple sequence alignment program ClustalX version 2.0.12 and alignment errors were corrected using BioEdit 7.2. The evolutionary history was inferred by using the Maximum Likelihood method and Le_Gascuel model [25]. Initial trees for the heuristic search were obtained automatically by applying Neighbor-Join and BioNJ algorithms to a matrix of pairwise distances estimated using the JTT model, and then selecting the topology with superior log likelihood value. A discrete Gamma distribution was used to model evolutionary rate differences among sites (4 categories (+*G*, parameter = 1.5010)). The rate variation model allowed for some sites to be evolutionarily invariable. There was a total of 8750 positions in the final dataset. Evolutionary analyses were conducted in MEGA 11 software [26,27].

### 2.3. %GC Content

The %GC content of all the sequences were calculated using Oligo calculator—an online tool of University of California Berkeley accessed at the URL http://mcb.berkeley.edu/labs/krantz/tools/oligocalc.html accessed on 18 November 2020.

## 3. Results

### 3.1. Overall Patterns of Horizontal Gene Transfer (HGT)

We analysed 221 *tnaA* gene sequences of all organisms retrieved from the NCBI database. As the presence of *tnaA* confers unique abilities to the host (particularly bacteria), we addressed the enduring question regarding the distribution and availability of *tnaA* across known taxonomic groups. Eighty-five prokaryote species have been reported to possess *tnaA* and/or its activity [5], and indole was considered to be synthesized exclusively by bacteria. Using a phylogenetic approach, we assessed the evolutionary patterns of *tnaA*. A total of 190 species, that is, 117 bacteria, 36 archaea, 16 fungi, and 21 eukaryotic species harboured the *tnaA* gene (Table 1). The nucleotide based trees were constructed to elucidate the diversity of the *tnaA* gene in the selected groups wherein the *tnaA* gene is susceptible to point mutations and can differentiate gene diversity in closely related organisms. The phylogenetic tree results were further corroborated using %GC content as a parametric method. Due to high variability in GC content of the prokaryotic genomes, internal factors such as GC parametric comparison can help infer horizontal gene transfers [28,29].

**Figure 1:**
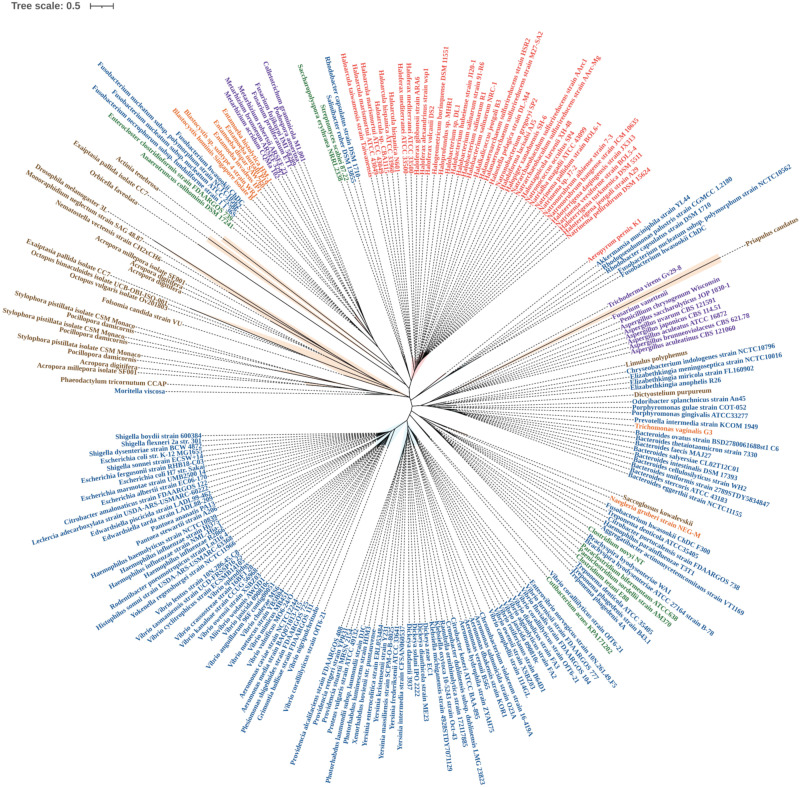
Nucleotide sequence phylogenetic tree (arbitrarily rooted Maximum Likelihood tree) of the *tnaA* gene representing a total of 221 nucleotide sequences belonging to 95 genera of various taxonomic groups. All the nodes represent bootstrap values ≥70 (1000 replications) and bootstrap value representation in Figure S1. Details of the phylogenetic analysis are provided in Methods. A complete list of genera and species and their taxonomic classifications are provided in Table 1. Species names are labelled according to phylogenetic classifications by the following colour codes. Gram-negative bacteria (blue), Gram-positive bacteria (green), archaea (red), fungi (purple), unicellular eukaryotes (orange), higher eukaryotes (brown).

In a phylogenetic reconstruction using a dataset containing the 221 *tnaA* nucleotides and their respective amino acid orthologs, γ-proteobacteria *tnaA* formed a statistically well-supported branch in both nucleotide and amino acid trees of Enterobacteriales and Vibrionales. Thus, it appears the order ‘Enterobacterales’, γ–proteobacteria have remained conserved in terms of *tnaA* gene sharing. We noticed several cases of the “non-vertical” appearance of *tnaA* in distantly related organisms including fungi and other unicellular eukaryotes (Figure 1). Phylogenetic analysis revealed that HGT of *tnaA* in eukaryotes was not the result of contamination. A wider distribution of *tnaA* was found in prokaryotes, particularly γ-proteobacteria. The diversity of *tnaA* followed phylogenetic relationships already established using unique features of rRNA sequences, according to which archaea, bacteria, and eukaryotes are segregated in isolated clades. However, *tnaA* based phylogeny revealed some random appearances of closely related gene sequences belonging to taxonomically diverse groups revealing HGTs (Figure 1).

**Figure 2:**
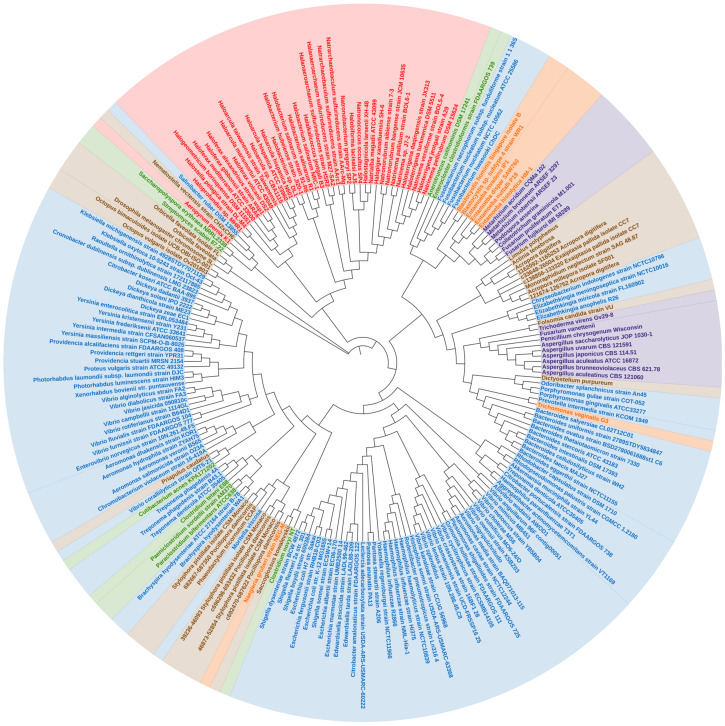
Amino acid sequence phylogenetic tree (arbitrarily rooted Maximum Likelihood tree) of the *tnaA* gene representing a total of 221 amino acid sequences belonging to 95 genera of various taxonomic groups. All the nodes represent bootstrap values ≥70 (1000 replications) and bootstrap value representation in Figure S2. Details of the phylogenetic analysis are provided in Methods. A complete list of genera and species and their taxonomic classifications are provided in Table 1. Species names are labelled according to phylogenetic classifications by the following colour codes. Gram-negative bacteria (blue), Gram-positive bacteria (green), archaea (red), fungi (purple), unicellular eukaryotes (orange), higher eukaryotes (brown).

The close sequence similarity of >73% for 99% sequence coverage and similar %GC content (Table S1) between *Yokenella* and *Rodentibacter tnaA* indicates a possibility of HGT between two distantly related organisms (Figure 1 and Figure 2), which belong to recently diverged sister orders *Enterobacteriales* and *Pasteurellales* of the class γ–proteobacteria, respectively. Previously, *glg* genes were found to undergo complex gene transfers among γ–proteobacteria and other main bacterial groups [30]. *Yokenella regensburgei* possess a *tnaA* gene sequence, which contrasts with reports that the organism is negative for indole production [31,32]. This apparent contradiction may be due to the presence of non-functional *tnaA*, and therefore needs more investigation. This is also observed in *Shigella.* sp strains which possess the *tnaA* gene but mutations in cluster 1 and cluster 3 of the *tnaA* operon consequently render them indole-negative [33]. On the other hand, *Enterococcus faecalis* and *Lactobacillus reuteri* produce indole (in vitro), which is indicative of *tnaA* activity (Table 1); however, the presence of *tnaA* within their genomes has yet to be determined. Recently, *Lactobacillus* sp. isolated from infant faeces were studied for their anti-obesity potential [34]. There is an inverse correlation between indole production and fat content which partially confirms functional presence of tryptophanase in *Lactobacillus* sp. [13].

### 3.2. TnaA Gene in Archaea

In archaea, the topology of *tnaA* phylogeny suggests a distinct but common bacterial ancestor (Figure 1 and Figure 2). Few thermophiles thrive within biofilms by secreting extracellular polymeric substances [35], and few halophiles that produce acyl-homoserine lactone (a quorum-sensing signal molecule) secrete extracellular polymeric substances and form biofilms (Table 2). Relationships among genera remained largely undisturbed. Sequences belonging to archaea—‘Halobacteriales, Haloferacales and Natrialbales’ belonging to the class Haloarchaea and other species formed a distinct clade, while the position of the thermophile ‘*Aeropyrum*’ shifted slightly (Figure 1) with varying %GC content from the archaeal cluster (Table 3, (a)). The exact benefit of indole production by archaea has yet to be determined. Although archaeal biofilms are poorly studied and characterized, increasing evidence suggests that like prokaryotes, archaea benefit from living in biofilms because they afford protection against environmental stresses, fluctuating pH, and toxic chemicals [36,37,38]. Furthermore, polymicrobial biofilms provide environments that facilitate the transfer of genetic material and enable syntrophic associations [35]. The appearance of *Aeropyrum pernix* in different clades reveals a distinct yet unknown origin, as it is the only representative thermophile that possesses *tnaA*. The proximity of the Natrialbales clade with those of Halobacteriales and Haloferacales indicates a common ancestry (Figure 1 and Figure 2). Indeed, *Haloterrigena* spp., which was previously categorized as a member of the Halobacteriaceae family, shows a close relationship with *Natrinema* spp. and was proposed to be included in the family *Natrialbaceae* [39] and supported by similar %GC content (Table S1). Interestingly, haloarchaea retained *tnaA* and other archaea lost the gene. *Salinibacter ruber* was found near the archaeal cluster (Figure 1) and has similar %GC content (Table 3, (b)) which further supports a common ancestry with halophilic characteristics [40]. *TnaA* is one of the genes extensively exchanged among members of the *Halobacteriaceae* and *Salinibacter* families. *Salinibacter ruber* was found to be indole-negative which is a common characteristic in most *Halobacteriaceae* species (Table 1) which further bolsters the case for common ancestry. Further study is required to decipher the influence of indole on the halophilic lifestyles of microbes and the survival strategies of the indole-negative biofilm-producing archaeal microbes.

### 3.3. Significance of tnaA in the Eukaryotic Life Cycle

For HGT in cases where a wider distribution of genes is found in donor and recipient lineages (and in other taxa), phylogenetic trees are used to investigate transfer polarity [41]. When a gene is only found in donor and recipient groups/taxa, it is generally assumed that the source of gene transfer must be the taxon displaying the most diverse representation of that gene, since the possibility of gene transfer in several organisms from a single species at the same time is unlikely [42]. HGT is an essential evolutionary tool and was thought to be limited to prokaryotes, but over the past decade, increasing evidence indicates genetic materials are exchanged between prokaryotes and eukaryotes (both endosymbionts and free-living organisms) [43,44,45]. The absence of *tnaA* in the genomes of vertebrates, including mammals, indicates the need for tryptophan degradation has reduced and that the function is largely provided by gut microbes as a result of co-evolution in a holobiont [46]. However, vertebrates have also adopted other means of tryptophan degradation via the kynurenine pathway by cleaving the indole moiety with tryptophan 2,3-dioxygenase in the liver [47].

It has been reported that about 145 genes in man originated from bacteria, including a few involved in amino-acid metabolism acquired by HGT [46]. For instance, Maximum Likelihood and Bayesian phylogenetic methods showed that eukaryotic pyruvate formate lyase, a homolog of the firmicutes gene, may have been acquired through HGT [48]. Similarly, firmicutes (*Anaerotruncus*, *Clostridium*, *Paraclostridium* and *Enterocloster*) were found within the cluster accommodating fungi and/or unicellular eukaryotes in the nucleotide sequence tree (Figure 1 and Figure 2). *TnaA* appears to have transferred from bacteria into fungi by two independent HGT events as evidenced by the formation of a distinct clade by members of Sordariomycetes and Eurotiomycetes (Figure 1 and Figure 2) corroborated by their similar %GC content (Table 3, (c)). Sordariomycetes (a clade of fungi—*Colletotrichum*, *Fusarium*, *Podospora*, and *Metarhizium*) appeared as a sister group of a *Blastocystis* branch (with high statistical support—based on bootstrap values) (Figure 1 and Figure 2) and similar %GC content (Table 3, (d)). Other related fungi belonging to the Ascomycota group (*Aspergillus*, *Penicillium*, and *Trichoderma*) also had a similar origin as their *tnaA* sequences were also found to be associated with a clade containing a few protozoa *Naegleria* and *Dictyostelium purpureum* (Figure 1). The abundance of several bacterial *tnaA* genes indicates that the origin of *tnaA* could be interleaved with members of different bacterial phyla, and endosymbiosis of *tnaA* possessing bacteria might be the reason for their common ancestry [43]. *E. histolytica tnaA* might be a result of HGT from a relative of the anaerobic bacterium *Fusobacterium* (Figure 1 and Figure 2) seconded with similar %GC content (Table 3, (e)). In contrast, the *T. vaginalis* appears to acquire the gene from a separate origin by HGT from a relative of the anaerobic *Bacteroides* group (Figure 1 and Figure 2) and similar %GC content (Table 3, (f)). Only three species of anaerobic protists, *Trichomonas vaginalis*, *Tritrichomonas foetus* and *E. histolytica* have been reported to produce indole [49,50].

HGT might have played a role in the evolution and symbiotic adaptation of various unicellular organisms, including eukaryotes. Most of the unicellular organisms (>30%) we studied are pathogenic, and in these organisms, *tnaA* provides unique advantages for living within a host [20,23]. Previously, Pfam-based analysis led to the identification of 18 HGT events from bacteria to *Dictyostelium*, which possesses two β-eliminating lyases [51]. Thus, it is evident that the transfer of genetic material occurs between prokaryotes and unicellular eukaryotes, and that these eukaryotes gain a competitive advantage. Indeed, genes of prokaryotic origin are commonly transferred multiple times between protists [43].

HGT events can be classified into two broad types; those that maintain pre-existing functions (maintenance transfers) and those that add new functionality, such as host interaction, defence and adaptation to extreme environments, to the recipient (innovative transfers) [52]. Of note, many lineages (e.g., amoebae, ciliates, dinoflagellates, and non-parasitic excavates) lack reports of HGT events, probably because of sampling bias. Fungi, especially Ascomycota, are prolific producers of indole alkaloids, many of which display potent biological activities [53]. However, the function of indole and its metabolites are still largely undetermined in fungi. The NCBI-BLAST sequence similarities between Fusarium (along with *Penicillium*, *Aspergillus*, and *Trichoderma*) and *Porphyromonas*, *Prevotella*, and *Trichomonas* exceed 60% with *e*-values of <3 × 10^−21^ and similar %GC content (Table S1), indicating a possible HGT from a similar prokaryotic ancestor of the order ‘Bacteroidales’.

Pyruvate can be produced during amino acid metabolism and is specifically required for the regeneration of NAD+ during anaerobic fermentation, whereas aerobic fermentation increases ATP generation via the TCA cycle [1,54,55]. The inclusion of *tnaA* in a genome broadens metabolic ability especially concerning carbon (gluconeogenesis), amino acid, and nitrogen metabolism [1]. The decomposition and grazing activities of protozoans contribute toward the nitrogen cycle via ammonia generation. Pyruvate and ATP may also be produced via other metabolic pathways and thus the addition of tryptophan conversion may merely result in HGT being considered a ‘maintenance transfer’. Bacteria and protists with such alternative pathways harbouring *tnaA* thereby gain additional biochemical activity that provides survival and competitive advantages. Tryptophan may also serve as an energy source in *S. salmonicida* due to the presence of three copies of a bacteria-like TnaA that generate pyruvate, indole, and NH_3_ from tryptophan [56]. ‘Indole’ plays several other roles in the ecosystem and is considered an ‘archetypical hormone’ as it can regulate the behaviours of prokaryotes [8] and eukaryotes, including higher vertebrates and plants [23]. Therefore, the production of indole by *tnaA* may provide a means of manipulating a neighbour’s behaviour, which is of particular use in the gut where bacteria dominate, and other taxonomically diverse groups must compete for nutrients. Thus, the ability to produce indole provides distinct advantages, which suggests horizontal *tnaA* transfer should be considered an ‘innovative transfer’. The transfer of genetic material in this way is considered an indispensable driver of the evolution of fungi dwelling in the gut of higher organisms [57]. In addition, fungi belonging to the Ascomycota phylum produce indole alkaloids, which have high bioactivities, and octopuses and other marine organisms can use indole to synthesize pigments (e.g., Tyrian purple). The muricid mollusc *Dicathais orbita* produces Tyrian purple with the help of tissue dwelling *Vibrio* sp. [18]

Tryptophan is an essential amino acid produced by the shikimic acid pathway (by bacteria and plants, but not animals). Indole is an intermediate of this pathway and may serve as a precursor for tryptophan synthesis [58]. Furthermore, the ability of *tnaA* to work reversibly gives credence to the notion that bacteria can modulate its ability to produce tryptophan or degrade it to pyruvate and indole. Interestingly, fungi and endophytes interact symbiotically with host plants by synthesizing indole acetic acid (a plant growth hormone).

**Table 1:** Organisms used in the study and their classification and indole production + positive; − negative; n/a—not available; v—varying.

Organism	Classification (Class; Order; and Family)	Indole Production	Reference	Organism	Classification (Class; Order; and Family)	Indole Production	Reference
				**Gram−negative bacteria**			
*A. caviae*	Gammaproteobacteria; Aeromonadales; Aeromonadaceae	+	[59]	*M. viscosa*	Gammaproteobacteria; Alteromonadales; Moritellaceae	−	[60]
*A. dhakensis*		+	[61]	*O. splanchnicus*	Bacteroidia; Bacteroidales; Odoribacteraceae	n/a	
*A. hydrophila*		+	[59]	*P. ananatis*	Gammaproteobacteria; Enterobacterales; Erwiniaceae	+	[62]
*A. media*		+	[59]	*P. stewartia*		+	[63]
*A. salmonicida*		+	[64]	*P. laumondii*	Gammaproteobacteria; Enterobacterales; Morganellaceae	−	[65]
*A. veronii*		+	[59]	*P. luminescens*		−	[65]
*A. actinomycetemcomitans*	Gammaproteobacteria; Pasteurellales; Pasteurellaceae	n/a		*P. shigelloides*	Gammaproteobacteria; Enterobacterales; Enterobacterales incertae sedis	+	[66]
*A. muciniphila*	Verrucomicrobiae; Verrucomicrobiales; Akkermansiaceae	n/a		*P. gingivalis*	Bacteroidia; Bacteroidales; Porphyromonadaceae	+	[67]
*A. wodanis*	Gammaproteobacteria; Vibrionales; Vibrionaceae	+	[68]	*P. gulae*		+	[69]
*B. cellulosilyticus*	Bacteroidia; Bacteroidales; Bacteroidaceae	n/a		*P. intermedia*	Bacteroidia; Bacteroidales; Prevotellaceae	+	[70]
*B. eggerthii*		+	[71]	*P. vulgaris*	Gammaproteobacteria; Enterobacterales; Morganellaceae	+	[72]
*B. faecis*		+	[73]	*P. alcalifaciens*	Gammaproteobacteria; Enterobacterales; Morganellaceae	+	[74]
*B. intestinalis*		+	[75]	*P. rettgeri*		+	[76]
*B. ovatus*		+	[73]	*P. stuartii*		+	[76]
*B. salyersiae*		+	[73]	*R. ornithinolytica*	Gammaproteobacteria; Enterobacterales; Enterobacteriaceae	+	[77]
*B. stercoris*		+	[78]	*R. capsulatus*	Alphaproteobacteria; Rhodobacterales; Rhodobacteraceae	n/a	
*B. thetaiotaomicron*		+	[73]	*R. palustris*	Alphaproteobacteria; Rhizobiales; Bradyrhizobiaceae	n/a	
*B. uniformis*		+	[71]	*R. pneumotropicus*	Gammaproteobacteria; Pasteurellales; Pasteurellaceae	+	[79]
*B. hyodysenteriae*	Spirochaetia; Brachyspirales; Brachyspiraceae	+	[80]	*S. ruber*	Bacteroidetes Order II. Incertae sedis; Rhodothermaceae	−	[81]
*C. violaceum*	Betaproteobacteria; Neisseriales; Chromobacteriaceae	+	[82]	*S. boydii*	Gammaproteobacteria; Enterobacterales; Enterobacteriaceae	−	[83]
*C. indologenes*	Flavobacteriia; Flavobacteriales; Weeksellaceae	−	[84]	*S. dysenteriae*		−	[33]
*C. amalonaticus*	Gammaproteobacteria; Enterobacterales; Enterobacteriaceae	n/a		*S. flexneri*		−	[33]
*C. koseri*		+	[85]	*S. sonnei*		−	[33]
*C. portucalensis*		−	[86]	*V. alginolyticus*	Gammaproteobacteria; Vibrionales; Vibrionaceae	+	[68]
*C.dublinensis*	Gammaproteobacteria; Enterobacterales; Enterobacteriaceae	+	[87]	*V. anguillarum*		v	[68]
*D. dadantii*	Gammaproteobacteria; Enterobacterales; Pectobacteriaceae	+	[88]	*V. campbellii*		+	[68]
*D. dianthicola*		+	[89]	*V. cholerae*		+	[90]
*D. solani*		+	[91]	*V. coralliilyticus*		+	[92]
*D. zeae*		+	[89]	*V. crassostreae*		+	[93]
*E. piscicida*	Gammaproteobacteria; Enterobacterales; Hafniaceae	+	[94]	*V. cyclitrophicus*		−	[68]
*E. tarda*		+	[94]	*V. diabolicus*		+	[95]
*E. anophelis*	Flavobacteriia; Flavobacteriales; Weeksellaceae	+	[96]	*V. fluvialis*		+	[68]
*E. meningoseptica*		+	[97]	*V. furnissii*		v	[98]
*E. miricola*		+	[97]	*V. jasicida*		+	[99]
*E. norvegicus*	Gammaproteobacteria; Vibrionales; Vibrionaceae	+	[100]	*V. kanaloae*		+	[93]
*E. albertii*	Gammaproteobacteria; Enterobacterales; Enterobacteriaceae	+	[101]	*V. lentus*		+	[68]
*E. coli*		+	[102]	*V. metoecus*		+	[103]
*E. fergusonii*		+	[104]	*V. mimicus*		+	[68]
*E. marmotae*		−	[105]	*V. nigripulchritudo*		+	[68]
*F. hwasookii*	Fusobacteria; Fusobacteriales; Fusobacteriaceae	+	[106]	*V. owensii*		+	[99]
*F. necrophorum*		+	[107]	*V. rotiferianus*		+	[99]
*F. nucleatum*		+	[106]	*V. splendidus*		+	[68]
*F. nucleatum*		+	[106]	*V. tasmaniensis*		+	[68]
*G. hollisae*	Gammaproteobacteria; Vibrionales; Vibrionaceae	+	[108]	*V. vulnificus*		+	[68]
*H. haemolyticus*	Gammaproteobacteria; Pasteurellales; Pasteurellaceae	+	[109]	*X. bovienii*	Gammaproteobacteria; Enterobacterales; Morganellaceae	n/a	
*H. influenzae*		+	[109]	*Y. enterocolitica*	Gammaproteobacteria; Enterobacterales; Yersiniaceae	+	[110]
*H. parainfluenzae*		+	[111]	*Y. frederiksenii*		+	[112]
*H. somni*	Gammaproteobacteria; Pasteurellales; Pasteurellaceae	+	[113]	*Y. intermedia*		+	[114]
*K. michiganensis*	Gammaproteobacteria; Enterobacterales; Enterobacteriaceae	+	[115]	*Y. kristensenii*		v	[116]
*K. oxytoca*		+	[117]	*Y. massiliensis*		+	[118]
*Ladecarboxylata*	Gammaproteobacteria; Enterobacterales; Enterobacteriaceae	+	[119]	*Y. regensburgei*	Gammaproteobacteria; Enterobacterales; Enterobacteriaceae	−	[31]
				**Gram−positive bacteria**			
*A. colihominis*	Clostridia; Clostridiales; Ruminococcaceae	+	[120]	*P. bifermentans*	Clostridia; Clostridiales; Peptostreptococcaceae	+	[121]
*C. novyi*	Clostridia; Clostridiales; Clostridiaceae	+	[122]	*S. erythraea*	Actinobacteria; Pseudonocardiales; Pseudonocardiaceae	n/a	
*C. tetani*		+	[123]	*S. scabiei*	Actinobacteria; Streptomycetales; Streptomycetaceae	n/a	
*C. acnes*	Actinobacteria; Propionibacteriales; Propionibacteriaceae	−	[124]	*T. denticola*	Spirochaetia; Spirochaetales; Spirochaetaceae	+	[125]
*E. clostridioformis*	Clostridia; Clostridiales; Lachnospiraceae	n/a		*T. phagedenis*		+	[126]
*P. sordellii*	Clostridia; Clostridiales; Peptostreptococcaceae	n/a					
				**Archaea**			
*A. pernix*	Thermoprotei; Desulfurococcales; Desulfurococcaceae	n/a		*H. larsenii*	Halobacteria; Halobacteriales; Halobacteriaceae	n/a	
*H. jeotgali*	Halobacteria; Halobacteriales; Halobacteriaceae	v	[127]	*H. pelagica*	Halobacteria; Halobacteriales; Halobacteriaceae	+	[128]
*H. sulfurireducens*	Halobacteria; Halobacteriales; Halobacteriaceae	n/a		*H. daqingensis*	Halobacteria; Natrialbales; Natrialbaceae	−	[129]
*H. hispanica*	Halobacteria; Halobacteriales; Haloarculaceae	v	[130]	*H. jeotgali*	Halobacteria; Natrialbales; Natrialbaceae Halobacteria; Natrialbales; Natrialbaceae	+	[131]
*H. marismortui*		−	[132]	*H. turkmenica*		+	[133]
*H. taiwanensis*		−	[134]	*Natrarchaeobaculum sulfurireducens*		−	[135]
*Haloarcula* sp.		−	[134]	*N. magadii*	Halobacteria; Natrialbales; Natrialbaceae	n/a	
*H. hubeiense*	Halobacteria; Halobacteriales; Halobacteriaceae	n/a		*N. pallidum*	Halobacteria; Natrialbales; Natrialbaceae	−	[136]
*H. salinarum*		v	[137]	*N. pellirubrum*	Halobacteria; Natrialbales; Natrialbaceae Halobacteria; Natrialbales; Natrialbaceae	−	[136]
*Halobacterium* sp.		n/a		*N. versiforme*		+	[138]
*H. lacisalsi*	Halobacteria; Natrialbales; Natrialbaceae	−	[139]	*Natrinema* sp.		v	
*H. alexandrinus*	Halobacteria; Haloferacales; Haloferacaceae	+	[140]	*N. gregoryi*		n/a	
*H. gibbonsii*		+	[130]	*N. occultus*	Halobacteria; Natrialbales; Natrialbaceae	n/a	
*H. mediterranei*		+	[141]	*N. aibiense*	Halobacteria; Natrialbales; Natrialbaceae	+	[142]
*H. volcanii*		+	[143]	*N. bangense*		+	[144]
*H. borinquense*	Halobacteria; Haloferacales; Haloferacaceae	+	[145]				
*H. xanaduensis*	Halobacteria; Halobacteriales; Halobacteriaceae	−	[146]				
				**Eukaryotes**			
		**Protists**					
*A. aculeatinus*	Eurotiomycetes; Eurotiales; Aspergillaceae	n/a		*N. gruberi*	Heterolobosea; Schizopyrenida; Vahlkampfiidae	n/a	
*A. aculeatus*		n/a		*T. vaginalis*	--; Trichomonadida; Trichomonadidae	+	[49]
*A. brunneoviolaceus*		n/a		*E. dispar*	Amoebozoa; Mastigamoebida; Entamoebidae Amoebozoa; Mastigamoebida; Entamoebidae	n/a	
*A. japonicus*		n/a		*E. histolytica*		+	[50]
*A. saccharolyticus*		n/a		*E. invadens*		n/a	
*A. uvarum*		n/a		*E. nuttalli*		n/a	
*C. graminicola*	Sordariomycetes; Glomerellales; Glomerellaceae	n/a					
*F. fujikuroi*	Sordariomycetes; Hypocreales; Nectriaceae	n/a		**Higher eukaryotes**			
*F. proliferatum*		n/a		*O. bimaculoides*	Cephalopoda; Octopoda; Octopodidae Cephalopoda; Octopoda; Octopodidae Anthozoa; Scleractinia; Acroporidae	n/a	
*F. vanettenii*		n/a		*O. vulgaris*		n/a	
*M. acridum*	Sordariomycetes; Hypocreales; Clavicipitaceae	n/a		*A. digitifera*	Anthozoa; Scleractinia; Acroporidae Anthozoa; Scleractinia; Pocilloporidae Anthozoa; Scleractinia; Acroporidae Anthozoa; Scleractinia; Pocilloporidae	n/a	
*M. brunneum*		n/a		*A. millepora*		n/a	
*M. robertsii*		n/a		*S. pistillata*	Anthozoa; Actiniaria; Aiptasiidae	n/a	
*P. chrysogenum*	Eurotiomycetes; Eurotiales; Aspergillaceae	n/a		*E. pallida*	Anthozoa; Actiniaria; Aiptasiidae	n/a	
*P. anserina*	Sordariomycetes; Sordariales; Chaetomiaceae	n/a		*A. tenebrosa*	Anthozoa; Actiniaria; Actiniidae	n/a	
*T. virens*	Sordariomycetes; Hypocreales; Hypocreaceae	n/a		*N. vectensis*	Anthozoa; Actiniaria; Edwardsiidae	n/a	
		*O. faveolata*	Anthozoa; Scleractinia; Merulinidae		n/a		
*M. neglectum*	Chlorophyceae; Sphaeropleales; Selenastraceae	n/a		*P. damicornis*	Anthozoa; Scleractinia; Pocilloporidae	n/a	
*D. purpureum*	Eumycetozoa; Dictyosteliales; Dictyosteliaceae	n/a		*S. kowalevskii*	Enteropneusta; --; Harrimaniidae [Hemichordata]	n/a	
*B. hominis,*	Bigyra; Opalinata; Blastocystidae	n/a		*L. polyphemus*	Merostomata; Xiphosura; Limulidae	n/a	
*Blastocystis* sp.		n/a		*F. candida*	Collembola; Entomobryomorpha; Isotomidae	n/a	
*P. tricornutum*	Bacillariophyceae; Naviculales; Phaeodactylaceae	n/a		*D. melanogaster*	Insecta; Diptera; Drosophilidae	n/a	
				*P. caudatus*	Priapulimorpha; Priapulimorphida; Priapulidae	n/a	

**Table 2:** List of archaea possessing the *tnaA* gene and/or indole producing activity.

Organism	Unique Growth Requirements	Indole		Biofilm (EPS or QS Signal)	References
		Gene (NCBI)	Production		
*Aeropyrum pernix* K1	Thermophile, 90 °C	+	−	−	[147]
*Halalkalicoccus jeotgali* B3	1.70–5.1 M NaCl	+	+	−	[131]
*Halanaeroarchaeum sulfurireducens* strain HSR2	3–5 M NaCl	+	NR	−	[148]
*Halanaeroarchaeum sulfurireducens* strain M27-SA2	3–5 M NaCl	+	NR	−	[149]
*Haloarcula hispanica* ATCC 33960	1.70–5.1 M NaCl	+	+	EPS	[132] [150]
*Haloarcula hispanica* N601		+	+/−	EPS	
*Haloarcula marismortui* ATCC 43049		+	−	EPS	
*Haloarcula taiwanensis* strain Taiwanensis		+	NR	NR	
*Haloarcula* sp. CBA1115		+			
*Halobacterium hubeiense* strain JI20-1		+			
*Halobacterium salinarum* NRC-1	3.5–5 M NaCl	+	NR	+	
*Halobacterium salinarum* R1	4.2 M NaCl	+	NR	EPS/AHL	
*Halobiforma lacisalsi* AJ5	>1.7 M NaCl	+	−	−	
*Haloferax alexandrinus* strain wsp1	4.3 M NaCl	+	+	−	[140]
*Haloferax gibbonsii* strain ARA6	1.7–4.3 M NaCl	+	+	EPS	
*Haloferax mediterranei* ATCC 33500	1–5.2 M NaCl	+	+	EPS	
*Haloferax volcanii* DS2	2.6–4.3 M NaCl	+	NR	+/AHLs	
*Halogeometricum borinquense* DSM 11551	3.4–4.3 M NaCl	+	+	+/AHLs	
*Halopiger xanaduensis* SH-6	4.3 M NaCl	+	−	−	
*Haloprofundus* sp. MHR1	0.9–4.8 M NaCl	+	−	−	
*Halostagnicola larsenii* XH-48	2.5–5.0 M NaCl	+	−	−	
*Halostella pelagica* strain DL-M4	2.6 M NaCl	+	NA	−	
*Haloterrigena daqingensis* strain JX313	1.7–5.5 M NaCl	+	−	−	
*Haloterrigena jeotgali* strain A29	2.6–3.4 M NaCl	+	+	−	
*Haloterrigena turkmenica* DSM 5511	2.6–3.4 M NaCl	+	NA	NA	
*Natrarchaeobaculum sulfurireducens* strain AArc1	3–5 M NaCl	+	−	NA	
*Natrialba magadii* ATCC 43099	3.4 M NaCl	+	NR	NA	
*Natrinema pallidum* strain BOL6-1	3.4–4.3 M NaCl	+	−	NA	
*Natrinema pellirubrum* DSM 15624	3.4–4.3 M NaCl	+	−	NA	
*Natrinema versiforme* strain BOL5-4	3.4–4.3 M NaCl	+	+	NA	
*Natronobacterium gregoryi* SP2	2.0–5 M NaCl	+	NR	NA	
*Natronococcus occultus* SP4	2 M NaCl	+	NR	AHL/Biofilm	
*Natronorubrum aibiense* strain 7-3	2.0–4.3 M NaCl	+	+	NA	[142]
*Natronorubrum bangense* strain JCM 10635	2.0–4.3 M NaCl	+	+	NA	[144]

NR: not reported; NA: not available; AHL: Acyl-homoserine lactone; EPS: extracellular polymeric substance. + positive; − negative.

**Table 3:** The %GC content comparison of (**a**) *Aeropyrum pernix K1* and archaeal cluster, (**b**) *Salinibacter ruber* and archaeal cluster, (**c**) Sadriomycetes and Euratiomycetes cluster, (**d**) Sadriomycetes and *Blastocystis.sp* branch, (**e**) *Fusobacterium* sp. branch and *Entamoeba histolytica* HM-1 and (**f**) *Bacteroides.sp* branch and *Trichomonas vaginalis* G3.

**(a)**		**(b)**		**(c)**	
**Organism**	**%GC content**	**Organism**	**%GC content**	**Organism**	**%GC content**
*N. occultus* SP4	68	*N. occultus* SP4	68	*A. aculeatinus* CBS 121060	65
*N. aibiense* strain 7-3	66	*N. aibiense* strain 7-3	66	*A. aculeatus* ATCC 16872	63
*N. gregoryi* SP2	65	*N. gregoryi* SP2	65	*A. brunneoviolaceus* CBS 621.78	65
*N. pallidum* strain BOL6-1	66	*N. pallidum* strain BOL6-1	66	*A. japonicus* CBS 114.51	62
*N. magadii* ATCC 43099	66	*N. magadii* ATCC 43099	66	*A. saccharolyticus* JOP 1030-1	65
*N. sulfurireducens* strain AArc1	67	*N. sulfurireducens* strain AArc1	67	*A. uvarum* CBS 121591	64
*H. borinquense* DSM 11551	63	*H. borinquense* DSM 11551	63	*P. anserina* S mat+	56
*H. gibbonsii* strain ARA6	68	*H. gibbonsii* strain ARA6	68	*M. acridum* CQMa 102	53
*H. jeotgali* strain A29	68	*H. jeotgali* strain A29	68	*M. brunneum* ARSEF 3297	52
*H. pelagica* strain DL-M4	67	*H. pelagica* strain DL-M4	67	*M. robertsii* ARSEF 23	53
*H. larsenii* XH-48	65	*H. larsenii* XH-48	65	*F. fujikuroi* IMI 58289	50
*H. xanaduensis* SH-6	69	*H. xanaduensis* SH-6	69	*F. proliferatum* ET1	50
*H. lacisalsi* AJ5	69	*H. lacisalsi* AJ5	69	*F. vanettenii*	58
*Halobacterium* sp. DL1	71	*Halobacterium* sp. DL1	71	*C. graminicola* M1.001	56
*H. salinarum* NRC-1	71	*H. salinarum* NRC-1	71		
*H. sulfurireducens* strain HSR2	67	*H. sulfurireducens* strain HSR2	67		
*H. jeotgali* B3	65	*H.jeotgali* B3	65		
*A. pernix* K1	56	*S. ruber* DSM 13855	67		
**(d)**		**(e)**		**(f)**	
**Organism**	**%GC content**	**Organism**	**%GC content**	**Organism**	**%GC content**
*P. anserina* S mat+	56	*F. hwasookii* ChDC	34	*B. cellulosilyticus* strain WH2	46
*M. acridum* CQMa 102	53	*F. hwasookii* ChDC F300	31	*B. eggerthii* strain NCTC11155	45
*M. brunneum* ARSEF 3297	52	*F. necrophorum* strain 1_1_36S	40	*B. faecis* MAJ27	46
*M. robertsii* ARSEF 23	53	*F. nucleatum* ATCC 25586	33	*B. intestinalis* DSM 17393	46
*F. fujikuroi* IMI 58289	50	*F. nucleatum* strain NCTC10562	33	*B. ovatus* strainBSD2780061688st1_C6	47
*F. proliferatum* ET1	50	*E. histolytica* HM-1	33	*B. salyersiae* CL02T12C01	46
*F. vanettenii*	58			*B. stercoris* ATCC 43183	44
*C. graminicola* M1.001	56			*B. thetaiotaomicron* strain 7330	48
*B. hominis*, Singapore isolate B	56			*T. vaginalis* G3	48
*Blastocystis* sp. strain WR1	57				

## 4. Conclusions

Among prokaryotes, we observed a wider distribution of *tnaA* in γ-proteobacteria, which share similar copies of the gene among themselves and therefore grouped. Our phylogenetic analysis suggests HGT has played a crucial role in *tnaA* gene transfer and provided ‘maintenance and innovative gain’ to recipients. These results are quite relevant from the host–parasite interaction perspective. Acquired *tnaA* has probably enabled unicellular eukaryotes to regulate the gut environment and live in synergy with microbiota and other enteric bacteria. We suggest that the adaptation of the intestinal protozoan *Blastocystis* sp. to the gut resulted in the lateral acquisition of the *tnaA* gene and enabled the supply of additional metabolites to the pre-existing metabolic systems.

Similarly, other unicellular eukaryotes such as *Dictyostelium*, *Entamoeba*, and *Trichomonas* may have inherited *tnaA* from a eukaryotic ancestor. However, the sparse presence of *tnaA* in higher eukaryotes (e.g., sea anemone, corals, octopuses, drosophila, and nematodes) is insufficient to reveal its origins. On the other hand, the common occurrence of *tnaA* in archaea belonging to the class Haloarchaea is intriguing and warrants further investigation of the role of indole in adaptation to halophilic environments. Collectively, these results reveal new avenues of research directed toward improved understanding of the roles of indole in various organisms including eukaryotes.

## Data Availability

The authors confirm that the data supporting the findings of this study are available within the article (and/or) its Supplementary Materials.

## Supplementary Materials

The following are available online at https://www.mdpi.com/article/10.3390/microorganisms9102156/s1, Table S1. % GC content of all the species sequences. Figure S1. Nucleotide sequence phylogenetic tree (arbitrarily rooted maximum likelihood tree) of the tnaA gene representing a total of 221 nucleotide sequences belonging to 95 genera of various taxonomic groups. All the nodes represent bootstrap values >= 70 (1000 replications). Details of the phylogenetic analysis are provided in Methods. A complete list of genera and species and their taxonomic classifications are provided in Table 1. Species names are labelled according to phylogenetic classifications by the following colour codes. Gram-negative bacteria (blue), Gram-positive bacteria (green), archaea (red), fungi (purple), unicellular eukaryotes (orange), higher eukaryotes (brown). Figure S2. Amino acid sequence phylogenetic tree (arbitrarily rooted maximum likelihood tree) of the tnaA gene representing a total of 221 amino acid sequences belonging to 95 genera of various taxonomic groups. All the nodes represent bootstrap values >= 70 (1000 replications). Details of the phylogenetic analysis are provided in Methods. A complete list of genera and species and their taxonomic classifications are provided in Table 1. Species names are labelled according to phylogenetic classifications by the following colour codes. Gram-negative bacteria (blue), Gram-positive bacteria (green), archaea (red), fungi (purple), unicellular eukaryotes (orange), higher eukaryotes (brown).
