# Supplementary material for: Diversity of the Tryptophanase Gene and Its Evolutionary Implications in Living Organisms
Source: Microorganisms. 2021 Oct 15;9(10):2156. doi: 10.3390/microorganisms9102156 (PMC8537960; doi:10.3390/microorganisms9102156)
Supplement: Supplementary file 1 [file microorganisms-09-02156-s001.zip › microorganisms-1382263-supplementary.pdf]

Supplementary material

Diversity of the tryptophanase gene and its evolutionary implications in living organisms

Bharath Reddy Boya, Prasun Kumar, Jin-Hyung Lee, and Jintae Lee*

School of Chemical Engineering, Yeungnam University, 280 Daehak-Ro, Gyeongsan 38541, Republic of Korea

***Corresponding author:**

E-mail: jtlee@ynu.ac.kr, Phone: +82-53-810-2533, Fax: +82-53-810-4631

Table S1. % GC content of all the species sequences

Organism	%GC content	Organism	%GC content
1162092-1165253 *Acropora digitifera*	42	*Histophilus somni* strain USDA-ARS-USMARC-63368	38
121674-126752 *Acropora digitifera*	44	*Klebsiella michiganensis* strain 4928STDY7071129	57
39236-46093 *Stylophora pistillata* isolate CSM Monaco	41	*Klebsiella oxytoca* 10-5243 strain Oct-43	56
46873-52854 *Stylophora pistillata* isolate CSM Monaco	39	*Leclercia adecarboxylata* strain USDA-ARS-USMARC-60222	51
682667-687350 *Pocillopora damicornis*	42	*Limulus polyphemus*	38
Acropora digitifera	41	*Metarhizium acridum* CQMa 102	53
Acropora millepora isolate SF001	39	*Metarhizium brunneum* ARSEF 3297	52
Acropora millepora isolate SF001	43	*Metarhizium robertsii* ARSEF 23	53
Actinia tenebrosa	39	*Monoraphidium neglectum* strain SAG 48.87	65
Aeromonas caviae strain NCTC12244	64	*Moritella viscosa*	40
Aeromonas dhakensis strain KOR1	65	*Naegleria gruberi* strain NEG-M	36
Aeromonas hydrophila strain ZYAH75	64	*Natrarchaeobaculum sulfurireducens* strain AArc1	67
Aeromonas media strain BAQ071013-115	64	*Natrarchaeobaculum sulfurireducens* strain AArc-Mg	67
Aeromonas salmonicida strain O23A	62	*Natrialba magadii* ATCC 43099	66
Aeromonas veronii B565	64	*Natrinema pallidum* strain BOL6-1	66
Aeropyrum pernix K1	56	*Natrinema pellirubrum* DSM 15624	68
Aggregatibacter actinomycetemcomitans strain VT1169	46	*Natrinema sp.* J7-2	66
Akkermansia muciniphila strain YL44	59	*Natrinema versiforme strain* BOL5-4	69

Aliivibrio wodanis AWOD1	41	*Natronobacterium gregoryi* SP2	65
Anaerotruncus colihominis DSM 17241	54	*Natronococcus occultus* SP4	68
Aspergillus aculeatinus CBS 121060	65	*Natronorubrum aibiense* strain 7-3	66
Aspergillus aculeatus ATCC 16872	63	*Natronorubrum bangense* strain JCM 10635	65
Aspergillus brunneoviolaceus CBS 621.78	65	*Nematostella vectensis* strain CH2xCH6	44
Aspergillus japonicus CBS 114.51	62	*Octopus bimaculoides* isolate UCB-OBI-ISO-001	38
Aspergillus saccharolyticus JOP 1030-1	65	*Octopus vulgaris* isolate Ov201803	40
Aspergillus uvarum CBS 121591	64	*Odoribacter splanchnicus* strain An45	43
Bacteroides cellulosilyticus strain WH2	46	*Orbicella faveolata*	41
Bacteroides eggerthii strain NCTC11155	45	*Orbicella faveolata*	41
Bacteroides faecis MAJ27	46	*Orbicella faveolata* isolate FL	40
Bacteroides intestinalis DSM 17393	46	*Paeniclostridium sordellii* strain AM370	32
Bacteroides ovatus strain BSD2780061688st1_C6	47	*Pantoea ananatis* PA13	48
Bacteroides salyersiae CL02T12C01	46	*Pantoea stewartii* strain A206	50
Bacteroides stercoris ATCC 43183	44	*Paraclostridium bifermentans* ATCC638	33
Bacteroides thetaiotaomicron strain 7330	48	*Penicillium chrysogenum* Wisconsin	51
Bacteroides uniformis strain 2789STDY5834847	53	*Phaeodactylum tricornutum* CCAP	48
Blastocystis hominis, Singapore isolate B	56	*Photorhabdus laumondii* subsp. laumondii strain DJC	40
Blastocystis sp. subtype 4 strain WR1	57	*Photorhabdus luminescens* strain HIM3	40
Brachyspira hyodysenteriae ATCC 27164 strain B-78	32	*Plesiomonas shigelloides* strain FDAARGOS_725	53
Brachyspira hyodysenteriae WA1	32	*Pocillopora damicornis*	43
c138856-133320 *Exaiptasia pallida* isolate CC7	38	*Podospora anserina* S mat+	56
c33648-26504 *Exaiptasia pallida* isolate CC7	39	*Porphyromonas gingivalis* ATCC33277	48
c692470-687622 *Pocillopora damicornis*	41	*Porphyromonas gulae* strain COT-052	49
Chromobacterium violaceum strain 16-419A	68	*Prevotella intermedia* strain KCOM 1949	48
Chryseobacterium indologenes strain NCTC10796	39	*Priapulus caudatus*	46

Citrobacter amalonaticus strain FDAARGOS_122	48	*Proteus vulgaris* strain ATCC 49132	37
Citrobacter koseri ATCC BAA-895	55	*Providencia alcalifaciens* strain FDAARGOS_408	41
Citrobacter portucalensis strain FDAARGOS_738	50	*Providencia rettgeri* strain YPR31	38
Clostridium novyi NT	31	*Providencia stuartii* MRSN 2154	40
Clostridium tetani E88	34	*Raoultella ornithinolytica* strain 172117885	56
Colletotrichum graminicola M1.001	56	*Rhodobacter capsulatus* strain DSM 1710	64
Cronobacter dublinensis subsp. dublinensis LMG 23823	56	*Rhodopseudomonas palustris* strain CGMCC 1.2180	64
Cutibacterium acnes KPA171202	59	*Rodentibacter pneumotropicus* strain Ln316_4	41
Dickeya dadantii 3937	59	*Saccharopolyspora erythraea* NRRL2338	71
Dickeya dianthicola strain ME23	59	*Saccoglossus kowalevskii*	45
Dickeya solani IPO 2222	59	*Salinibacter ruber* DSM 13855	67
Dickeya zeae EC1	57	*Shigella boydii* strain 600384	50
Dictyostelium purpureum	32	*Shigella dysenteriae* strain BCW_4872	50
Drosophila melanogaster chromosome 3L	47	*Shigella flexneri* 2a str. 301	50
Drosophila melanogaster chromosome 3R	48	*Shigella sonnei* strain ECSW+14	51
Edwardsiella piscicida strain LADL99-462	57	*Streptomyces scabiei* 87.22	72
Edwardsiella tarda strain LADL88-209	55	*Stylophora pistillata* isolate CSM Monaco	43
Elizabethkingia anophelis R26	39	*Treponema denticola* ATCC 35405	41
Elizabethkingia meningoseptica strain NCTC10016	40	*Treponema phagedenis* 4A	45
Elizabethkingia miricola strain FL160902	39	*Treponema phagedenis* strain B43.1	45
Entamoeba dispar SAW760	34	*Trichoderma virens* Gv29-8	46
Entamoeba histolytica HM-1	33	*Trichomonas vaginalis* G3	48
Entamoeba invadens IP1	41	*Vibrio alginolyticus* strain FA2	47
Entamoeba nuttalli P19	33	*Vibrio anguillarum* 96F contig00051	47
Enterocloster clostridioformis strain FDAARGOS_739	44	*Vibrio campbellii* strain 1114GL	49

Enterovibrio norvegicus strain 10N.261.49.F5	52	*Vibrio cholerae* MS6	48
Escherichia albertii strain EC06-170	49	*Vibrio coralliilyticus* strain OfT6-21	48
Escherichia coli O157:H7 str. Sakai	51	*Vibrio crassostreae* strain 16BF1_28	47
Escherichia coli str. K-12 MG1655	51	*Vibrio cyclitrophicus* strain ECSMB14105	46
Escherichia fergusonii strain RHB18-C03	51	*Vibrio diabolicus* strain FA3	47
Escherichia marmotae strain UMB2500_14	50	*Vibrio fluvialis* strain FDAARGOS_104	54
Folsomia candida strain VU	42	*Vibrio furnissii* strain FDAARGOS_777	55
Fusarium fujikuroi IMI 58289	50	*Vibrio jasicida* 090810c	47
Fusarium proliferatum ET1	50	*Vibrio kanaloae* strain CCUG 56968	46
Fusarium vanettenii	58	*Vibrio lentus* strain 10N.286.45.C8	47
Fusobacterium hwasookii ChDC	34	*Vibrio metoecus* strain YB5B04	48
Fusobacterium hwasookii ChDC F300	31	*Vibrio mimicus* MB451	48
Fusobacterium necrophorum subsp. funduliforme strain 1_1_36S	40	*Vibrio nigripulchritudo*	50
Fusobacterium nucleatum subsp. nucleatum ATCC 25586	33	*Vibrio owensii* strain XSBZ03	48
Fusobacterium nucleatum subsp. polymorphum strain NCTC10562	33	*Vibrio rotiferianus* strain B64D1	48
Grimontia hollisae strain FDAARGOS_111	52	*Vibrio splendidus*	47
Haemophilus haemolyticus strain NCTC10839	40	*Vibrio tasmaniensis* strain UCD-FRSSP16_25	47
Haemophilus influenzae R2866	40	*Vibrio vulnificus* MO6-24/O	50
Haemophilus influenzae strain Hi375	40	*Xenorhabdus bovienii* str. puntauvense	43
Haemophilus influenzae strain NML-Hia-1	40	*Yersinia enterocolitica* strain ERL053484	48
Haemophilus parainfluenzae T3T1	39	*Yersinia frederiksenii* ATCC 33641	48
Halalkalicoccus jeotgali B3	65	*Yersinia intermedia* strain CFSAN060537	49
Halanaeroarchaeum sulfurireducens strain HSR2	67	*Yersinia kristensenii* strain Y231	49
Halanaeroarchaeum sulfurireducens strain M27-SA2	67	*Yersinia massiliensis* strain SCPM-O-B-8025	49
Haloarcula hispanica ATCC 33960	64	*Yokenella regensburgei* strain NCTC11966	44

Haloarcula hispanica N601	64	*Halopiger xanaduensis* SH-6	69
Haloarcula marismortui ATCC 43049	63	*Haloprofundus sp.* MHR1	68
Haloarcula sp. CBA1115	64	*Halostagnicola larsenii* XH-48	65
Haloarcula taiwanensis strain Taiwanensis	64	*Halostella pelagica* strain DL-M4	67
Halobacterium hubeiense strain JI20-1	71	*Haloterrigena daqingensis* strain JX313	67
Halobacterium salinarum NRC-1	71	*Haloterrigena jeotgali* strain A29	68
Halobacterium salinarum R1	71	*Haloterrigena turkmenica* DSM 5511	69
Halobacterium salinarum strain 91-R6	71	*Haloferax gibbonsii* strain ARA6	68
Halobacterium sp. DL1	71	*Haloferax mediterranei* ATCC 33500	63
Halobiforma lacisalsi AJ5	69	*Haloferax volcanii* DS2	68
Haloferax alexandrinus strain wsp1	68	*Halogeometricum borinquense* DSM 11551	63

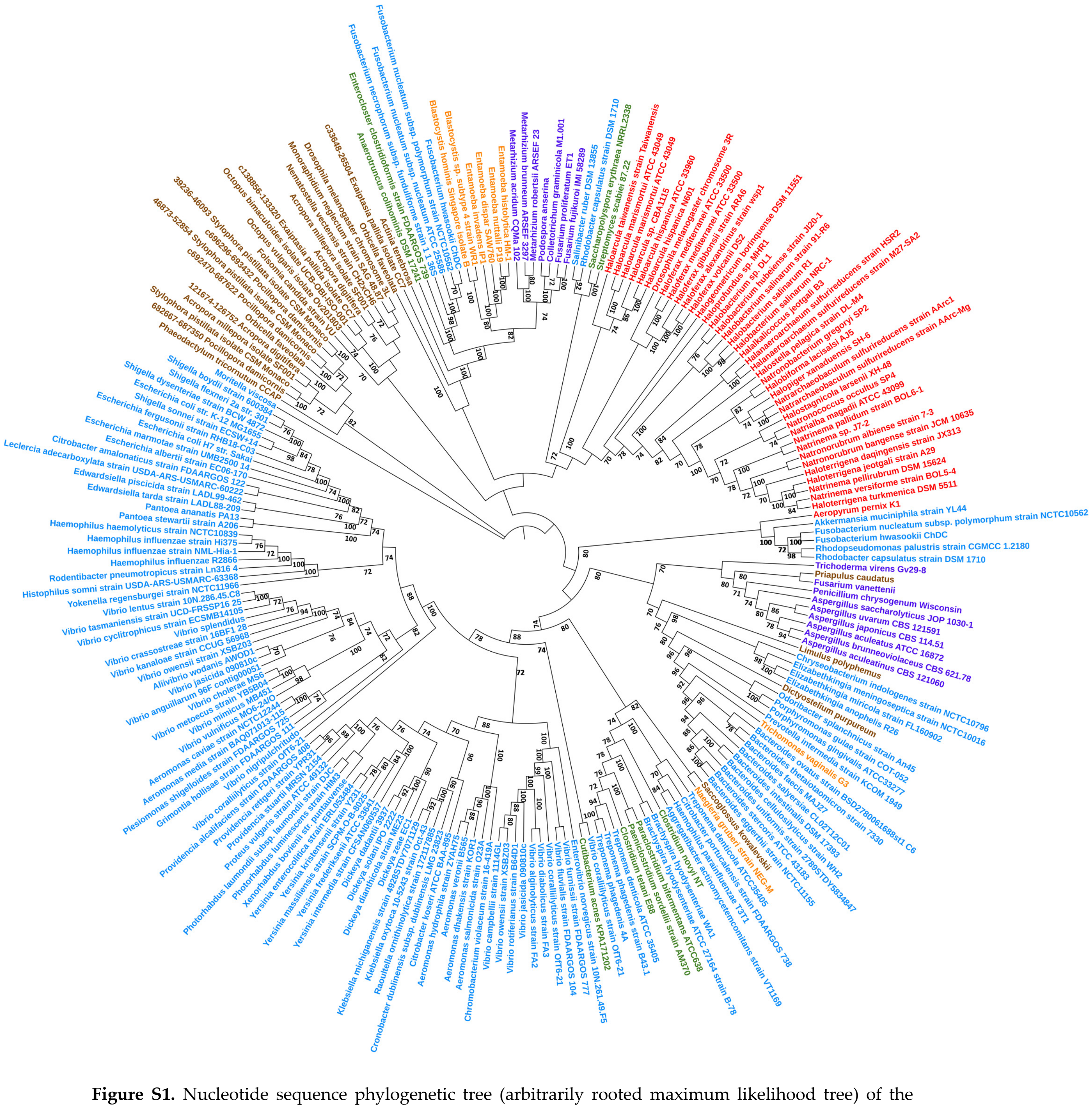

Figure S1. Nucleotide sequence phylogenetic tree (arbitrarily rooted maximum likelihood tree) of the *tnaA* gene representing a total of 221 nucleotide sequences belonging to 95 genera of various taxonomic groups. All the nodes represent bootstrap values >=70 (1000 replications). Details of the phylogenetic analysis are provided in Methods. A complete list of genera and species and their taxonomic classifications are provided in Table 1. Species names are labelled according to phylogenetic classifications by the following colour codes. Gram-negative bacteria (blue), Gram-positive bacteria (green), archaea (red), fungi (purple), unicellular eukaryotes (orange), higher eukaryotes (brown).

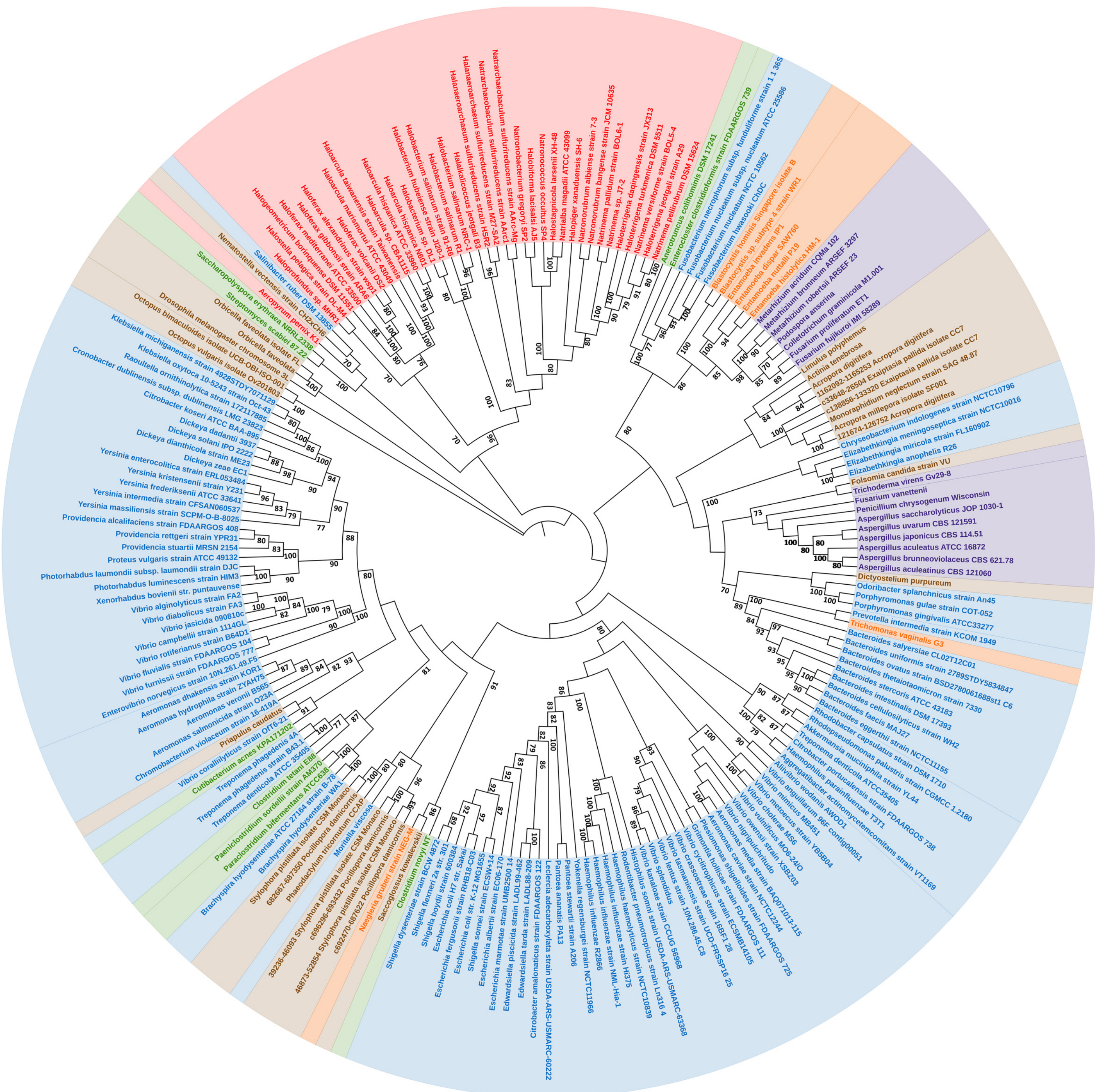

Figure S2. Amino acid sequence phylogenetic tree (arbitrarily rooted maximum likelihood tree) of the *tnaA* gene representing a total of 221 amino acid sequences belonging to 95 genera of various taxonomic groups. All the nodes represent bootstrap values >=70 (1000 replications). Details of the phylogenetic analysis are provided in Methods. A complete list of genera and species and their taxonomic classifications are provided in Table 1. Species names are labelled according to phylogenetic classifications by the following colour codes. Gram-negative bacteria (blue), Gram-positive bacteria (green), archaea (red), fungi (purple), unicellular eukaryotes (orange), higher eukaryotes (brown).
